# Effective RNA Knockdown Using CRISPR-Cas13a and Molecular Targeting of the *EML4-ALK* Transcript in H3122 Lung Cancer Cells

**DOI:** 10.3390/ijms21238904

**Published:** 2020-11-24

**Authors:** Saifullah, Matomo Sakari, Takeshi Suzuki, Seiji Yano, Toshifumi Tsukahara

**Affiliations:** 1 Area of Bioscience and Biotechnology, School of Materials Science, Japan Advanced Institute of Science and Technology (JAIST), 1-1 Asahidai, Nomi City, Ishikawa 923-1292, Japan; saifullah@jaist.ac.jp (S.); m-sakari@jaist.ac.jp (M.S.); 2 Division of Functional Genomics, Cancer Research Institute, Kanazawa University, Kakuma-Machi, Kanazawa 920-1192, Japan; suzuki-t@staff.kanazawa-u.ac.jp; 3 Molecular Therapeutic Target Research Unit, Institute for Frontier Science Initiative, Kanazawa University, Kakuma-Machi, Kanazawa 920-1192, Japan; 4 Division of Medical Oncology, Cancer Research Institute, Kanazawa University, Takara-Machi, Kanazawa 920-0934, Japan; syano@staff.kanazawa-u.ac.jp; 5 Nano Life Science Institute, Kanazawa University, Kakuma-Machi, Kanazawa 920-1192, Japan; 6 Division of Transdisciplinary Science, Japan Advanced Institute of Science and Technology (JAIST), 1-1 Asahidai, Nomi City, Ishikawa 923-1292, Japan

**Keywords:** RNA stability, RNA knockdown, CRISPR-Cas13a, firefly luciferase, EML4-ALK, cell viability

## Abstract

RNAi technology has significant potential as a future therapeutic and could theoretically be used to knock down disease-specific RNAs. However, due to frequent off-target effects, low efficiency, and limited accessibility of nuclear transcripts, the clinical application of the technology remains challenging. In this study, we first assessed the stability of Cas13a mRNA and guide RNA. Next, we titrated Cas13a and guide RNA vectors to achieve effective knockdown of firefly luciferase (FLuc) RNA, used as a target transcript. The interference specificity of Cas13a on guide RNA design was next explored. Subsequently, we targeted the *EML4-ALK* v1 transcript in H3122 lung cancer cells. As determined by FLuc assay, Cas13a exhibited activity only toward the orientation of the crRNA–guide RNA complex residing at the 5′ of the crRNA. The activity of Cas13a was maximal for guide RNAs 24–30 bp in length, with relatively low mismatch tolerance. After knockdown of the *EML4-ALK* transcript, cell viability was decreased up to 50%. Cas13a could effectively knock down FLuc luminescence (70–76%), mCherry fluorescence (72%), and EML4-ALK at the protein (>80%) and transcript levels (26%). Thus, Cas13a has strong potential for use in RNA regulation and therapeutics, and could contribute to the development of personalized medicine.

## 1. Introduction

RNAi technology has gained wide acceptance as a tool for knocking down specific cellular transcripts; hence it has been valuable for elucidation of molecular pathways, factors involved in diseases, and potential treatments [1,2,3,4]. However, many issues must be addressed before this technology can be used in clinical practice, including off-target effects, the requirement for multiple rounds of transfection to achieve effective knockdown, the limited ability to target nuclear transcripts, and the costs associated with making chemical modifications in siRNA or generating viral vectors for shRNA [1,5,6,7,8]. Hence, alternative methods for effective knockdown of RNA remain constrained.

DNA manipulation tools such as CRISPR-Cas9 enable researchers to dissect the functions of particular genetic elements [9,10,11], and such tools can also bind and cleave RNA in a programmable manner [12]. One concern about using Cas9 in DNA or RNA engineering is that it retains the ability to interact with nontargeted DNA, potentially leading to unintended permanent mutations in the genome [13]. In light of that consideration, the discovery of Cas13 (class II type VI RNA-guided RNA-targeting CRISPR-associated Cas effector) [14,15,16,17,18], which can only target RNA, has opened new opportunities in the field of RNA regulation and therapeutics that are not available when using conventional RNAi or DNA-targeting CRISPR tools.

The *EML4* and *ALK* genes are located 12 Mb apart on chromosome 2 (2p21 and 2p23), but are oriented in different directions. The genes can be fused through paracentric inversion, resulting in development of lung adenocarcinoma [19]. At least 15 variants of the oncogenic fusion have been identified to date [20]. All variants contain the basic region of EML4, which is required for the activation of ALK through oligomerization and autophosphorylation [19,20]. Upon activation, ALK plays a primary role in the development of lung adenocarcinoma, as well as colorectal and breast cancers [21]. The fusion variant of highest frequency is *EML4-ALK* v1 [20], which is present in the H3122 cell model. To date, inhibitors like crizotinib have demonstrated significant antitumor activity against these cancers, but eventual resistance limits the therapeutic benefits [22,23,24]. Therefore, a new therapeutic strategy is needed for the treatment of lung adenocarcinoma.

In this study, we examined CRISPR-Cas13a and crRNA–guide RNA from the standpoint of stability, effective knockdown efficiency, orientation of crRNA–guide RNA complex, and interference specificity on guide RNA design using firefly luciferase as a model target. Next, we used the programmable knockdown capacity of the Cas13a machinery to target *ALK* RNA in *EML4-ALK* fusion transcripts in H3122 lung cancer cells, with the goal of decreasing cell viability and providing proof-of-principle of a novel therapeutic approach.

## 2. Results

### 2.1. Analysis of RNA Stability

**Figure 1:**
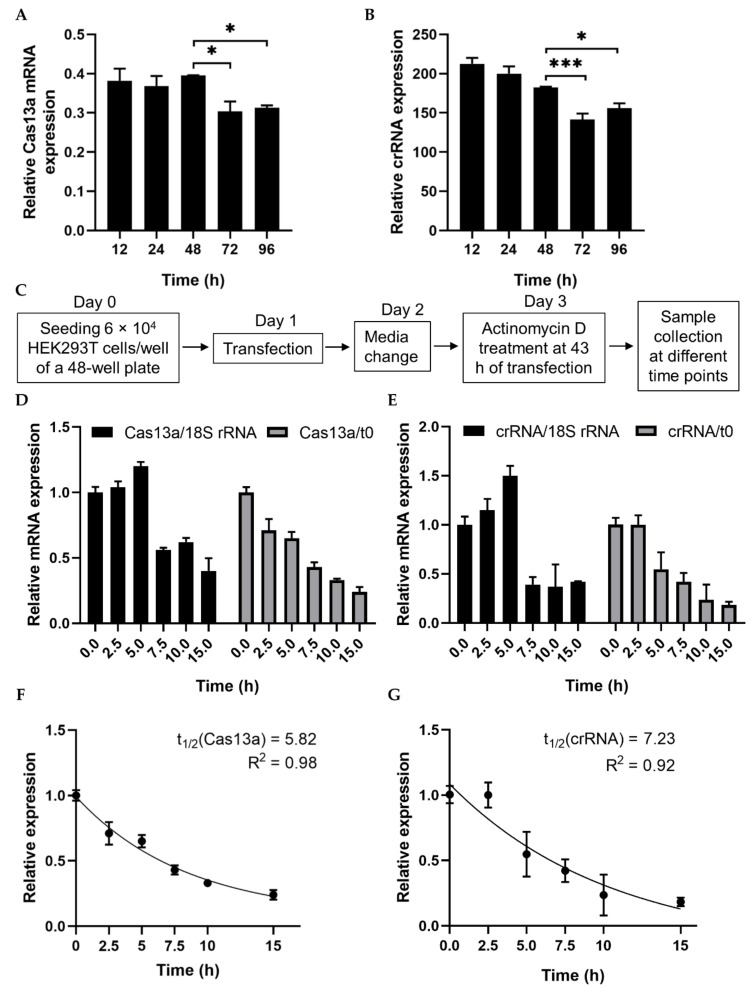
Analysis of RNA stability using a transcription inhibitor. (**A**,**B**) RT-PCR quantification of Cas13a and crRNA–guide RNA transfected into HEK293T cells. Total RNA was isolated after 12, 24, 48, 72, or 96 h, and subjected to cDNA synthesis and RT-PCR. Data were normalized against *GAPDH*. (**C**) Schematic of treatment with the transcription inhibitor actinomycin D (ActD) for half-life analysis. (**D**) qRT-PCR of Cas13a mRNA abundance after treatment with ActD for the indicated times. Cas13a mRNA levels were normalized against the corresponding levels of *18S rRNA* and against time point 0 (i.e., no ActD treatment). (**E**) qRT-PCR result of crRNA–guide expression after treatment with ActD for the indicated times. mRNA levels were normalized as in (**D**). (**F**) Exponential one-phase decay model of Cas13a mRNA fitted to a time course of transcriptional inhibition, normalized against the t0 time point. (**G**) One-phase decay model of crRNA–guide RNA fitted to a time course of transcriptional inhibition, normalized against the t0 time point. All data represent mean values ± SEM from three individual experiments. qRT-PCR results represent three biological replicates and two technical replicates. Statistical significance was determined by Student’s *t*-test. *** *p*  <  0.001, * *p*  <  0.05.

mRNA turnover is a key determinant of the abundance of cellular transcripts and, in turn, the level of protein or functional RNA. We first checked the stability of Cas13a mRNA and crRNA–guide RNA to characterize their decay rates and expression pattern. Both plasmid DNAs were transfected separately into HEK293T cells, and expression started to decrease 48 h after transfection (Figure 1A,B, Figure S1B). To calculate the decay rates, we estimated the RNA level at various time points after treatment with the transcriptional inhibitor actinomycin D (ActD). RNA abundance was determined in the absence of ActD treatment (t0) and after treatment. RNA abundance at various time points was initially normalized against a relatively stable housekeeping gene, human *18S rRNA*, rather than *GAPDH* [25], but inhibition was not linear (Figure 1C–E). Since ActD inhibits both RNA pol II and III, we subsequently normalized against t0. To determine the RNA decay rate, we fitted an exponential one-phase decay model to the normalized expression data. The half-lives of Cas13a and the crRNA–guide RNA were calculated as 5.82 and 7.23 h, respectively (Figure 1F,G). These data will be useful in future experiments involving Cas13a.

### 2.2. Optimizing the Quantity of Cas13a for Efficient RNA Knockdown

**Figure 2:**
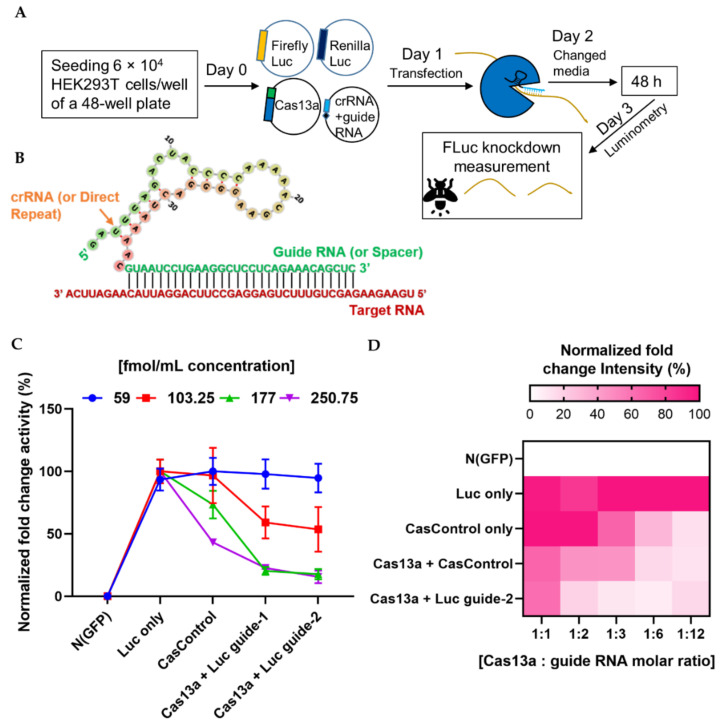
Quantitative analysis of Cas13a and guide RNA. (**A**) Experimental outline. (**B**) Schematic of the strategy for design of the crRNA–guide RNA complex targeting a specific RNA. (**C**) Quantity of Cas13a plasmid required for sufficient knockdown: line graph shows four concentrations (59, 103.25, 177, and 250.75 fmol/mL) of Cas13a vector. N (GFP), negative control; Luc only, luciferase expression without Cas13a; CasControl, Cas13a with nontarget guide RNA; Cas13a + Luc guide-1/2, Cas13a with target guide-1/2. The FLuc reporter was used as a target transcript while RLuc was used as an internal control. In this experiment, 177 fmol/mL Cas13a yielded sufficient knockdown (68% and 76% in guide-1 and -2), although a small reduction was also observed in CasControl (177: CasControl); (**D**) Quantity of crRNA–guide RNA plasmid required for knockdown. Heat map shows five different amounts of guide RNAs with 177 fmol/mL Cas13a (1:1, 1:2, 1:3, 1:6, and 1:12). Left side: N (GFP), negative control; Luc only, luciferase expression without Cas13a; CasControl only, nontarget guide without Cas13a; Cas13a + CasControl, Cas13a with nontarget guide; and Cas13a + Luc guide-2, Cas13a with target guide-2. The target transcript and internal control were used as (**C**). Where a 1:3 (Cas13a: guide RNA) molar ratio was considered satisfactory. All data represent mean values ± SEM from three individual experiments.

We next investigated the ability of the programmable endonuclease Cas13a to knock down firefly luciferase (FLuc) mRNA in HEK293T cells. First, we optimized the quantities of Cas13a and guide RNA for knockdown of FLuc (where *Renilla* luciferase, RLuc, was used as an internal control). A lower concentration (59 fmol/mL) of Cas13a was not sufficient to decrease luciferase luminescence (Figure 2C), but a higher concentration (177 fmol/mL) substantially decreased the level of luciferase RNA relative to CasControl (68% for guide-1 and 76% for guide-2). Excess Cas13a (250.75 fmol/mL) dramatically affected overall luciferase expression. We observed similar luminescence activity when we titrated the guide RNA (Figure 2D). Hence, we used 177 fmol/mL Cas13a concentration with a 1:3 molar ratio of guide RNA in subsequent knockdown experiments. It is worth noting that this quantity was not optimal for all Cas13a experiments, and merely provides an estimate of the amount of RNA that is required for sufficient Cas13a activity. Optimization of the absolute and relative quantities of Cas13a and guide RNA quantity is important before starting a new experiment.

### 2.3. Optimal Condition for RNA Knockdown and Preference of 5′ crRNA for Cas13a Activity

**Figure 3:**
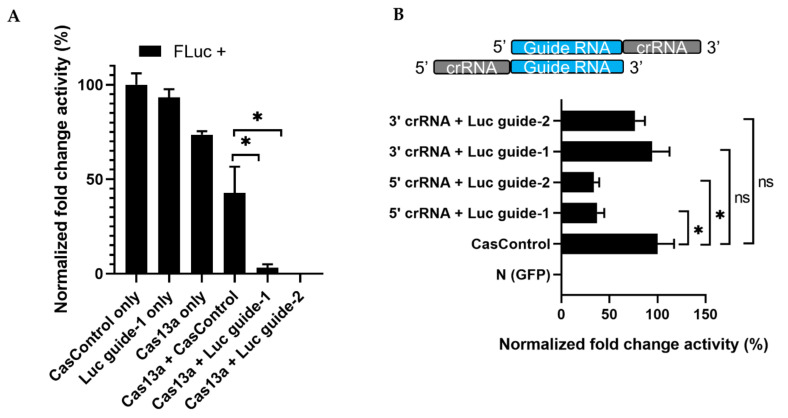
RNA knockdown and preference of Cas13a functionality for 5′ crRNA–guide RNA. (**A**) Knockdown of FLuc: FLuc +, presence of firefly luciferase during transfection; CasControl only, nontargeting guide only; Luc guide-1 only, guide RNA for target FLuc; Cas13a only, Cas13a plasmid without any guide; Cas13a + CasControl, Cas13a plasmid and nontargeting guide plasmid transfection; Cas13a + Luc guide-1/2, targeted guide-1/2 and Cas13a. The FLuc was used as a target transcript while RLuc was used as an internal control. (**B**) Preference of Cas13a functionality for 5′ crRNA–guide RNA complex: N (GFP), negative control; CasControl, nontargeting guide RNA; 5′/3′crRNA + Luc, Cas13a with 5′ or 3′ crRNA–guide RNA target (FLuc) guide-1/2. The target transcript and internal control were same as (**A**). All data represent mean values ± SEM from three individual experiments. Statistical significance was measured by Student’s *t*-test. * *p* < 0.05, ns, not significant.

Next, to observe the effect of Cas13a, crRNA, and experimental integrity, we transfected individual factors in the presence or absence of FLuc, using the optimized Cas13a and guide RNA concentrations described above. No luminescence was observed in the absence of FLuc. Luminescence was decreased 70% and 76% relative to CasControl in FLuc guide-1 and -2, respectively, in the presence of FLuc (Figure 3A). However, we also observed a reduction when Cas13a vector alone was used, potentially due to an effect of Cas13a on cellular metabolic load. Subsequently, we sought to determine which RNA strand/direction of the crRNA–guide RNA complex resulted in greater Cas13a ribonuclease activity. Ribonuclease activity exhibited a significant preference for the 5′ crRNA–guide RNA site (63% and 67% knockdown for guide-1 and -2, respectively) over the 3′ crRNA–guide RNA site (6% and 24% reduction; guide-1 and -2) relative to CasControl (Figure 3B). Given that the crRNA scaffold is important for the effective nuclease activity of Cas13a, we used 5′ crRNA–guide RNA in subsequent experiments.

### 2.4. Selectivity of Cas13a in Guide RNA Design

**Figure 4:**
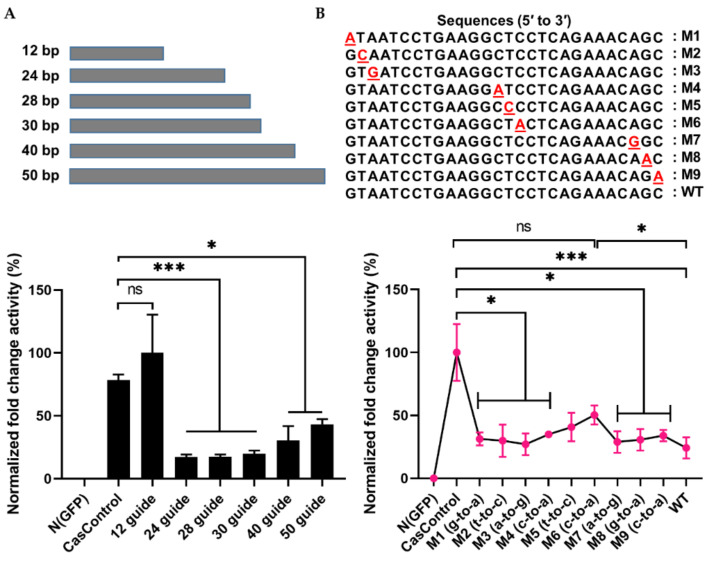
Effect of guide RNA design on Cas13a. (**A**) Length of guide RNA: 12, 24, 28, 30, 40, and 50 bp guide RNAs were tested; knockdown started at a length of 24 bp. The FLuc was used as a target transcript while RLuc was used as an internal control to obtain the guide RNA length limit. (**B**) Sensitivity of Cas13a to a single–base mismatch in the guide RNA: M1–M3, mismatch at the 5′ end; M4–M6, mismatch in the middle seed region; M7–M9, a mismatch at the 3′ end. Red letter indicates mismatch nucleotide. Cas13a activity was sensitive to mismatches at all sites, especially in the middle region. Values are normalized against CasControl and expressed as percentages. The target reporter transcript and internal control were the same as (**A**). All data represent mean values ± SEM from three individual experiments. Statistical significance was determined by Student’s *t*-test. *** *p*  <  0.001, * *p*  <  0.05. ns, not significant.

To address the interference specificity of Cas13a on guide RNA design, we first explored the effect of guide RNA length on Cas13a knockdown activity using 12, 24, 28, 30, 40, and 50 bp guide RNAs (in other words, spacer RNAs) (see Figure 2B and Figure 4A). As shown in Figure 4A, Cas13a was unable to achieve knockdown with a 12 bp guide, but significantly decreased luciferase levels with a 24, 28, or 30 bp guide (>70%); its activity decreased with longer guide RNAs (40 or 50 bp). To further investigate the dependence of Cas13a activity on guide RNA design, we introduced a single–base pair mismatch in the 5′, middle, or 3′ region of a 28 bp guide RNA. Relative to CasControl, luciferase luminescence was inhibited 68–70%, 58%, and 76% by guide RNAs with a mismatch in the 5′ or 3′ region, a mismatch in the middle region, and no mismatch, respectively (Figure 4B: M1–M3, 5′ site; M4–M6, middle region; M7–M9, 3′ site). Thus, Cas13a does not allow a mismatch in the guide RNA, particularly in the middle region. These results will aid in application of Cas13a to disruption of disease-relevant RNAs.

### 2.5. Validation of Effective Knockdown: mCherry and Molecular Targeting of EML4-ALK Oncofusion Genes

We speculated that the endonuclease activity of Cas13a would vary among target transcripts. With this in mind, we targeted mCherry mRNA in HEK293T cells, and found that mCherry fluorescence was diminished up to 72% (Figure S2). In addition, we targeted *ALK* in the *EML4-ALK* fusion oncogene in the H3122 cell line model for human lung adenocarcinoma.

**Figure 5:**
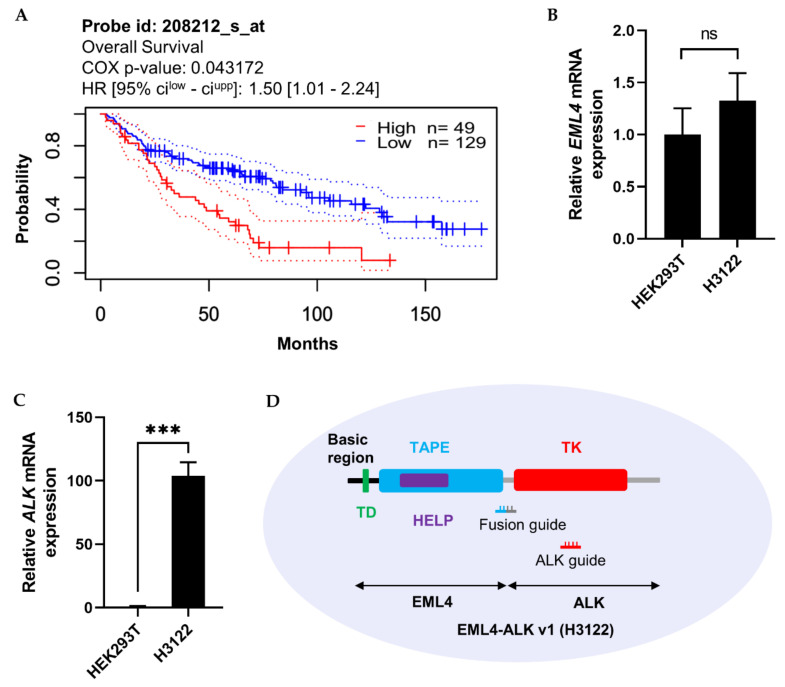
Clinical importance of *ALK* expression in lung adenocarcinoma, and *EML4* and *ALK* mRNA expression in HEK293T and H3122 cells. (**A**) Kaplan–Meier analysis of patient survival in lung adenocarcinoma. Data were retrieved from the PrognoScan cancer microarray database, with COX *p*-value < 0.05. The prognostic curve differentiates lung adenocarcinoma patients with high (red) and low (blue) *ALK* expression. The dotted lines indicate the maximum and minimum values of the survival average. (**B**,**C**) mRNA levels (assessed by qRT-PCR) of *EML4* and *ALK* in HEK293T and H3122 cells. *GAPDH* was used as an internal control gene. (**D**) Graphical representation of EML4-ALK v1 oncofusion with the associated functional domain and guide RNA location in H3122 cells. Left side: TD, trimerisation domain; TAPE, tandem atypical propeller domain; HELP, hydrophobic motif in EML proteins; and TK, tyrosine kinase domain. Fusion guide, *EML4-ALK* target guide at the fusion point of *EML4* and *ALK*; ALK guide, *EML4-ALK* target guide in the TK domain. qRT-PCR results represent three biological replicates and two technical replicates. Statistical significance was determined by Student’s *t*-test. *** *p*  <  0.001. ns, not significant.

The importance of the *ALK* expression pattern for prognosis of lung adenocarcinoma patients was determined based on information in the PrognoScan microarray database, using a threshold COX *p*-value of 0.05. As shown in Figure 5A, higher expression of *ALK* was inversely proportional to patient survival. Moreover, *ALK* not only fuses with *EML4* but also forms genetic fusions with other mRNA partners such as *STRN*, *TPM1*, *PPP4R3B*, *ACTG2*, *STK39*, *KCNQ5*, *KCNQ5*, *MALAT1*, *GTF2IRD1*, *GALNT14*, and *SCEL*, which, according to data retrieved from the TCGA PanCancer Atlas using cBioPortal (10,953 patients/10,967 samples), contribute to the development of eight other cancer types (Table 1). Therefore, regulation of *ALK* mRNA represents a potential therapeutic strategy.

More than 15 variants of *EML4-ALK* fusions have been reported. The most common is *EML4-ALK* v1 (E13; A20) [20]. We compared the expression levels of *EML4* and *ALK* between H3122 and a nonfusion cell line, HEK293T (Figure 5B,C). The results revealed that *EML4* was not detectably expressed, but *ALK* was expressed 100-fold more highly in H3122 than in HEK293T. Hence, we designed two guide RNAs against the fusion point and N-terminal region of ALK (Figure 5D) to knock down the *ALK* gene in cells expressing the *EML4-ALK* fusion, with the goal of determining whether ALK inhibition has an influence on cellular physiology.

**Figure 6:**
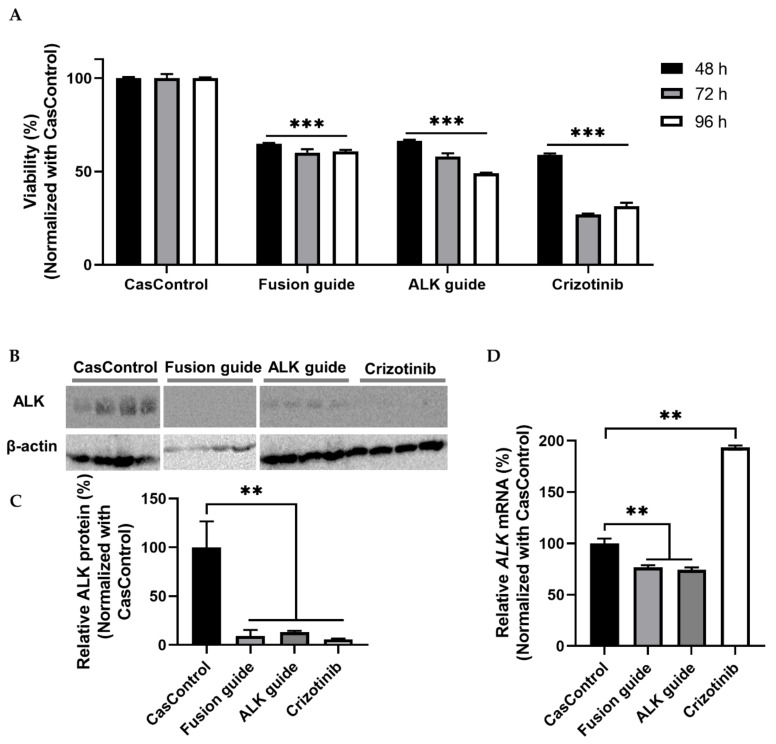
Cell viability and EML4-ALK knockdown in H3122 cells. (**A**) Cell viability (determined by MTT assay) after knockdown in H3122 cells at 48, 72, and 96 h after transfection. CasControl, nontargeting guide; Fusion guide, *EML4-ALK* target guide in the fusion point; ALK guide, *EML4-ALK* target guide in the tyrosine kinase (TK) domain; Crizotinib, positive control drug. (**B**) Western blotting of ALK knockdown. H3122 cells treated with Cas13a and guide RNA or crizotinib for 72 h. CasControl, Cas13a with nontarget guide; Fusion guide and ALK guide, *EML4-ALK* target guide RNAs; Crizotinib, positive control drug. β-actin was used as a loading control. (**C**) Representative graph of western blot gel image shown in (**B**). (**D**) qRT-PCR results of *ALK* mRNA knockdown in H3122 cells treated as in **B**. All data represent mean values ± SEM from three, four, or six individual experiments. qRT-PCR results represent three biological replicates and two technical replicates. Statistical significance was determined by Student’s *t*-test. *** *p*  <  0.001, ** *p*  <  0.01.

*ALK* knockdown resulted in 34–51% inhibition of cell viability at 48, 72, and 96 h after transfection relative to CasControl (Figure 6A). This observation indicates that Cas13a significantly inhibited the viability of H3122 cells. Next, we confirmed that ALK knockdown at the protein level was greater than 80% (Figure 6B,C). qRT-PCR revealed that knockdown at the transcript level was 23–26% (Figure 6D). Taken together, the cell viability and knockdown results indicated that the Cas13a effector effectively knocked down the fusion oncogene *EML4-ALK* in the H3122 lung cancer cell line. To check the reproducibility of *EML4-ALK* knockdown using Cas13a, we found considerable inhibition of cell viability and similar degree of ALK protein knockdown in H2228 cells expressing *EML4-ALK* v3a/b oncofusion gene (Figure S3).

## 3. Discussion

We observed that the mRNA expression of Cas13a and crRNA–guide RNA started to decrease 48 h after transfection in HEK293T cells. The half-lives of Cas13a and crRNA–guide RNA were 5.82 and 7.23 h, respectively (Figure 1). A previous study reported that expression of Cas13a mRNA decreases 2 h after transfection in glioma cells [26], however, those authors did not fuse Cas13a with msfGFP, which may explain the short expression time. As previously reported, msfGFP increases the stability of Cas13a, which we used in our fusion [15,16]. The authors of the glioma study checked expression levels but did not quantify the mRNA decay rate for Cas13a or crRNA–guide RNA.

Abudayyeh and colleagues discovered CRISPR/Cas13a and extensively studied the system in bacterial, human, and plant cells [14,15,16]. They used 150 ng of Cas13a and 300 ng of guide RNA vectors for *Cypridina* luciferase transcript knockdown in HEK293FT cells of a 96-well plate (40% or 72–75% knock down). Our optimization experiments revealed that 177 fmol/mL Cas13a vector (equivalent to 187.5 ng in a 96-well plate) and a 1:3 molar ratio of vector-to-guide RNA were sufficient to achieve 70–76% knockdown of FLuc mRNA (Figure 2 and Figure 3A). When we used double the amount of guide RNA vectors, we observed knockdown in both the control and target samples (Figure 2D; 1:6 and 1:12 molar ratio), potentially due to off-target effects or an overload of guide RNA. Our target transcript was firefly luciferase RNA, which is 27–29% similar to the *Gaussia* and *Cypridina* luciferases used by Abudayyeh et al. (Figure S1C). Konermann et al. used 200 ng Cas13d and 200 ng guide RNA vector to knock down mCherry mRNA (87–92%) in HEK293FT cells [17], similar to the amounts used in this study. We believe that it is preferable to titrate the guide RNA vector concentration based on the molar ratio rather than the amount of vector, as Cas13a is large, but the guide RNA is very short. For instance, if we were to add 300 ng guide RNA with 150 ng Cas13a, the concentration of guide RNA would be too high, and the excess foreign RNA could increase the cellular metabolic load. Therefore, titration of Cas13a and guide RNA vectors before performing a new experiment is important for obtaining high efficiency of knockdown.

From the standpoint of Cas13a activity, we found that it was best for the crRNA (direct repeat) to be 5′ of the guide RNA (spacer) (Figure 3B). This is consistent with previous studies of Cas13a (LwaCas13 or LshCas13a), Cas13c, and Cas13d [14,15,16,17,27]. By contrast, the optimal relative position is different in the Cas13b system, which prefers the crRNA to be 3′ of the guide RNA [13,16,27]. The stem–loop structure of crRNA and their order are important for the functionality of Cas13a, and the ideal order differs among Cas effector types.

To understand Cas13a’s target RNA binding and cleavage activity, we explored the length constraints for guide RNAs in human HEK293T cells. We found that a 24–30 bp guide RNA was sufficient for maximal FLuc mRNA knockdown (>75%)—cleavage activity was reduced when the guide was shorter (12 bp) or longer (40 or 50 bp) (Figure 4A). A similar pattern (21/24 bp length) was observed in a previous study that used an in vitro assay [15]. However, with one exception observed in human cells, previous work showed that shortening a guide RNA below 28 bp decreases Cas13a activity [15,16]. By contrast, we observed maximal activity between 24 and 30 bp. The reason for this discrepancy might be related to differences in experimental handling, cell types, or target genes. Cas13a cleavage activity was never observed with 40 or 50 bp guide RNAs. Next, we observed that Cas13a activity was sensitive to mismatches between the guide RNA and target RNA transcript (Figure 4B). Previous studies of the RNA cleavage specificities of Cas13a and Cas13b in HEK293FT cells, using either crRNA spacers or target RNA plasmids containing single or double mismatches, reported mismatch sensitivity, particularly in the middle portion of the guide RNA (seed region) [15,16]. We also noticed such a sensitivity in the middle regions of three guide RNAs (Figure 4B; M4-M6 guide RNAs). This sensitivity feature increases the applicability of Cas13a to target transcripts, especially for knockdown of disease-relevant RNA. Recently one in vitro study on Cas9 and Cas12a suggested that the tolerance of mispairing between target DNA and guide RNA is unexpectedly high and significantly influenced by polarity of the target DNA strand [28]. Which could generate unintended permanent error through collateral genomic activity in human genome [28]. Similarly, Cas13 also showed collateral cleavage activity in vitro assay [14,29]. Fascinatingly, so far, this collateral activity of Cas13 has not been detected in either human or plant cell lines [15,16]. Further experiment is needed to comprehend the Cas13′s collateral cleavage activity in the border context of immunity.

Cas13a activity varies among target transcripts [15,16,17]. In this study, we were able to decrease mCherry mRNA expression up to 72% using Cas13a (Figure S2), whereas another study reported 92% knockdown of mCherry using a different CRISPR effector (Cas13d) [17]. To further confirm the programmable endonuclease activity of Cas13a, we performed experiments in the H3122 and H2228 lung cancer cell lines. Information from the PrognoScan database indicated that higher expression of ALK is significantly correlated with lower patient survival in lung adenocarcinoma (Figure 5A), suggesting that if we could decrease ALK expression, patient survival rate might be improved. This is because, inhibition of EML4-ALK with an ALK tyrosine kinase inhibitor like TAE684, or via knockdown using RNAi results in the retraction of downstream signaling and induction of apoptosis primarily through RAS-MAPK signaling in *EML4-ALK*-positive cells [30,31,32]. However, the cancer eventually develops resistance to inhibitors [31,32], thereby limiting clinical benefits. On the other hand, RNAi has higher off-target effect, and limited accessibility to nuclear transcript [15,16]. Therefore, we used Cas13a-mediated RNA knockdown approach to regulate the expression of *EML4-ALK* oncofusion transcript in this study.

Katayama et al. achieved around fifty percent cell viability and robust ALK knockdown (at protein level) using siRNA in H3122 cells and H3122 CR (crizotinib resistance) cells [22]. Here, we confirmed up to 50% cell viability (determined by MTT assay in H3122 cells) and over 80% ALK knockdown based on western blot analyses (H3122 and H2228 cells) (Figure 6 and Figure S3). In contrast, one study succeeded to reduce viability over 60% using the siRNA technique [33]. Initial response of crizotinib to cell viability (69–74%) is greater than Cas13a-mediated RNA knockdown (34–51%) in this study (Figure 6A) and other TKI studies [22,32,33]. The reduction in cell viability that we observed and the efficiency of EML4-ALK knockdown indicated that Cas13a-mediated targeting of the *EML4-ALK* transcript not only knocked down ALK expression but also changed cell physiology, as reflected by reductions in cell viability at three different time points (Figure 6). This leads us to conclude that RNA-mediated gene knockdown using CRISPR/Cas13a is effective, low-cost, long-lasting, and programmable.

Despite the remarkable advantages of Cas13a, it has some drawbacks. Cas13a still retains some off-target effects [16] and protospacer flanking site (PFS) preference [26], and has lower efficiency than counterparts such as Cas13b or Cas13d, but it is still superior to RNAi from the standpoints of specificity and efficiency [15,16,17]. Off-target effects must be avoided, as even a single nonspecific knockdown could change a vital function in the target cells. Hence, new RNA-guided CRISPR or other RNA cleavage tools are still needed to resolve these issues and enable the development of therapeutics based on this technology. In this study, we have not yet determined the apoptotic and cell proliferative effects. We will conduct these phenomena to define whether ALK knockdown leads to anti-proliferative or apoptotic effects or both of them. Although ALK is an orphan receptor, knockdown can decrease the expression of normal ALK (nonfusion) function. Our fusion-guided RNA can solve this issue because it only binds to the fusion point of *EML4-ALK* oncogene. More rational guide design aimed at inhibiting expression of the *EML4-ALK* transcript has potential as an RNA-based therapy.

## 4. Materials and Methods

### 4.1. Plasmid Vectors

Firefly luciferase pGL3-Promoter Vector (GenBank Accession Number: U47298) and *Renilla* luciferase pRL-TK Vector (GenBank Accession Number: AF025846) were purchased from Promega (Madison, WI, USA). pRSET-B mCherry was a gift from Dr. Hidekazu Tsutsui, JAIST, Japan. mCherry DNA was amplified from pRSET-B mCherry by PCR and inserted into pCS2+ vector at the *Bam*HI and *Eco*RI sites. Plasmid LwCas13a-msfGFP was purchased from Addgene (plasmid #91902) [15]. The Cas13a-GS-msfGFP region (insert) was cloned into the pcDNA3-EGFP (Addgene; plasmid #13031) by homology assembly cloning. An *Xho*I restriction site, a Kozak sequence, and an NLS were inserted at the 5′ site, and an NLS, 2 × Myc tag, TAA stop codon, and *Xba*I restriction site were inserted at the 3′ site adjacent to the Cas13a-GS-msfGFP insert. Oligo sequences used for mCherry, Cas13a, and pcDNA3 amplifications are listed in Table S1.

PCRs were performed in a total volume of 25 µL, consisting of 10 µL Milli-Q water, 12.5 µL KOD One master mix (TOYOBO, Osaka, Japan), 0.75 µL (10 pmol) of each oligo, and 1 µL (5 ng/µL) template plasmid. PCR conditions were as follows: preheating at 95 °C for 2 min, 17 cycles of denaturation at 98 °C for 10 s, annealing at 60 °C for 5 s; extension at 68 °C for 1 min, and final extension at 72 °C for 7 min. Next, the vector PCR product was treated with *Dpn*I: 25 µL PCR product, 9.5 µL Milli-Q water, and 0.5 µL *Dpn*I (NEB, Ipswich, MA, USA). PCR products were electrophoresed on 1% agarose gels and purified using the Gel PCR purification kit (MACHERY-NAGEL GmbH & Co. KG, Düren, North Rhine-Westphalia, Germany). The assembly reaction contained 1 µL vector PCR product, 3 µL insert PCR product (1:3), and 4 µL HiFi assembly master mix (NEB). The final vector sequence was confirmed by Sanger sequencing (see Supplementary Materials). The vector map is shown in Figure S1A.

### 4.2. Guide RNA Design

The guide RNAs were designed as the reverse complement of the target cDNA site to occupy the Cas13a machinery. The sequences were selected as follows: PFS on target A, T, or C; GC content 40–60%, off-target effects minimized by the BLAST tool in NCBI, and RNA secondary structure checked using the RNAfold web server (http://rna.tbi.univie.ac.at/cgi-bin/RNAWebSuite/RNAfold.cgi) to avoid self-complementarity and complex secondary structure. Following design, the cDNA sequences of crRNA (direct repeat) and guide RNA (spacer) with 5′ *Bam*HI and 3′ *Eco*RI overhangs (Fwd strand 5′ GATCC, 3′ G; Rev strand 5′ AATTC, 3′ G) on both strands were ordered as oligos from Eurofins Genomics, Tokyo, Japan. The oligos were annealed in a thermocycler. Reactions were performed in a total volume of 30 µL, consisting of 15 µL Milli-Q water, 4 µL (10 pmol) of each oligo, and 7 µL of 2 × ligation buffer (Promega). Annealing conditions were as follows: 95 °C for 5 min, decreased 7 °C every 5 min to 32 °C, 25 °C for 10 min, 4 °C for ∞ min. The annealed fragments were then ligated with *Bam*HI- and *Eco*RI-digested pCS2+, and cloned into *E. coli* DH5 alpha competent cells. Construct was validated by restriction digestion and Sanger sequencing. Sequences of guide RNAs and crRNAs are provided in Table S2.

### 4.3. Sanger Sequencing

Sanger sequencing was performed using a 3130XL Genetic Analyzer (Applied Biosystems, Japan) as described in our previous protocol [34]. In some cases, 500–800 ng samples of plasmids with specific primers (Table S3) were sent to a company (Eurofins Genomics) for DNA sequencing.

### 4.4. Cell Culture and Transfection

HEK293T cells (RIKEN Cell Bank, Japan) were cultured in Dulbecco’s modified Eagle’s medium (DMEM, Nacalai Tesque, Kyoto, Japan) supplemented with 10% fetal bovine serum (COSMO Bio, Brazil) at 37 °C in a humidified incubator under an atmosphere containing 5% CO_2_. Unless otherwise noted, cells were plated in 48-well plates at 6 × 10^4^ cells/well; cell counts were determined using a hemocytometer. The next day, cells were transfected with the indicated vectors using PEI reagent. H3122 and H2228 cells were kindly provided by Dr. Jeffrey A Engelman (Novartis Institutes for Biomedical Research, Cambridge, MA, USA) [35,36]. Both cells were cultured in RPMI 1640 (Nacalai Tesque) medium supplemented with 10% FBS and transfected using the Lipofectamine 3000 Reagent (Invitrogen, Carlsbad, CA, USA).

### 4.5. RNA Stability Assay

To monitor the expression of Cas13a mRNA and crRNA–guide RNAs, 2.5 × 10^3^ HEK293T cells were seeded per well; transfections were performed on the following day. Total RNA was extracted after 12, 24, 48, 72, and 96 h, and subjected to cDNA synthesis and RT-PCR. For half-life measurements, 6 × 10^3^ HEK293T cells were seeded per well. The next day, 375 ng each of Cas13a and crRNA–guide RNA vector were transfected separately. Culture media was replaced the following day. On the third day, for time point 0 (t0), cells were washed with D-PBS (Nacalai Tesque), lysed with 100 µL TRIzol reagent (Invitrogen), and then kept in a −80 °C freezer. For the remaining time points (2.5, 5, 7.5, 10, and 15 h), cells were simultaneously treated with 5 µg/mL ActD (final concentration). Total RNA was isolated from the cells and subjected to cDNA synthesis and qRT-PCR. The C_T_ value was normalized against human *18S rRNA* and the corresponding value at t0. Half-life was estimated from t0-normalized data.

The half-life of a transcript was calculated as follows: half-life = ln(2)/kdecay [37]. This equation could be fit to the data using nonlinear regression (exponential one-phase decay; least square), which is available in GraphPad Prism.

### 4.6. Total RNA Extraction

Cells were washed quickly with D-PBS and lysed by addition of 100 µL/well TRIzol reagent (Invitrogen). The lysed samples were homogenized by vortexing for 2–3 min. Next, 25 µL chloroform was added to the solution and mixed by hand for 15 s. The sample was centrifuged at 12,000× *g* for 15 min at 4 °C, and about 50 µL supernatant was transferred to a new tube. Next, 62.5 µL of 100% isopropanol was added to the supernatant, mixed by inversion, and incubated at room temperature for 10 min. The samples were centrifuged at 12,000× *g* for 10 min at 4 °C, and the supernatant was discarded. The pellet was washed with 200 µL of 80% ethanol, and the supernatant was discarded. The pellet was then air-dried at room temperature for 5–10 min and dissolved in Milli-Q water.

### 4.7. cDNA Synthesis

cDNA synthesis was performed in a total volume of 20 µL. Reaction mixtures contained 100 ng RNA, 0.5 µL of each oligo (dT) primer and random primer, 1 µL of 10 mM dNTPs, and water to a final volume of 14 µL. After mixing, the sample was incubated at 65 °C for 5 min and cooled on ice for 5 min. Next, 4 µL of 5 × buffer, 1 µL of ReverTra Ace (TOYOBO), and 1 µL of 0.1 M DTT were added to the solution, followed by mixing. Incubation was performed in a thermocycler (GeneAmp PCR System 9700, Applied Biosystems, Singapore) as follows: 50 °C for 30 min and 55 °C for 30 min for synthesis, 70 °C for 15 min for inactivation, and 4 °C hold.

### 4.8. RT-PCR Quantification

PCR reactions were performed in a total volume of 20 μL on a thermocycler (GeneAmp PCR System 9700). Reaction mixtures contained 9.3 μL of H_2_O, 1 μL cDNA template, 4 μL of 5 × Green GoTaq Flexi buffer (Promega), 2 μL of 25 mM MgCl_2_, 2 μL of 2 mM dNTPs, 0.1 μL Taq polymerase (Promega), and 0.8 μL (10 pmol) of each primer. Sequences of all primers are provided in Table S4. PCR conditions were as follows: pre-heating at 95 °C for 3 min, 25 cycles of 95 °C for 30 s, 56 °C for 30 s, and 72 °C for 90 s, and final extension at 72 °C for 7 min. *GAPDH* was used as a loading control. Two-microliter aliquots of each PCR product was loaded on 6% polyacrylamide gels. The ImageJ software was used to quantify band intensities [38].

### 4.9. SYBR Green-Based qPCR

SYBR Green–based qPCR was performed in a total volume of 10 μL on an MX 3000P STRATAGENE Multiplex Quantitative PCR System (Agilent Technologies, USA). The reactions consisted of 3.5 μL water, 5 μL TB Green, 0.2 μL of 50 × ROX reference dye II (TaKaRa Bio, Japan), 0.5 μL cDNA template, and 0.8 μL (10 pmol) of each primer; sequences of all primers are provided in Table S4. qPCR conditions were as follows: pre-heating at 95 °C for 3 min, 45 cycles of 95 °C for 15 s, 55 °C for 10 s, and 72 °C for 15 s, and a final cycle of 95 °C for 1 min, 55 °C for 30 s, and 95 °C for 30 s. The C_T_ values were normalized against *GAPDH* or *18S rRNA* expression using the 2^−ΔΔCT^ method [39].

### 4.10. Luciferase Luminometry Assay

Bioluminescence of luciferase was measured according to the Dual-Luciferase Reporter (DLR) Assay System (Promega) protocol with minor modifications. Briefly, HEK239T cells were seeded at 6 × 10^4^ cells/well of a 48-well, and cotransfected with firefly luciferase (as a target) and *Renilla* luciferase (as an internal control) at a 1:10 ratio with Cas13a and guide RNA vectors. Forty-eight hours after transfection, the media were removed, and the cells were washed with D-PBS. The cells were partially lysed in a 50 μL/well of 1 × passive lysis buffer (PLB) (Promega, USA) and incubated for 15 min at room temperature with shaking. Next, the plate was kept on ice, and luminescence was measured in each well. First, a blank was measured: 10 µL of 1 × PLB was mixed into 30 μL luciferase substrate I (LARII) in a measuring tube, firefly relative light unit (RLU) was observed on a Gene Light 55 (Microtech Nichion Co., Ltd., Japan), 30 µL Stop and Glo reagent was added to the same tube, and *Renilla* RLU was observed. Second, the sample was measured: 2 μL cell lysate and 8 μL of 1 × PLB were mixed into 30 μL LARII, and then the RLU values were observed as described above for the blank. The blank value was subtracted from all sample values. The condition of the machine was set to dual-luciferase assay mode: first delay time, 3 s; first count time, 6 s; second delay time, 3 s; second count time, 6 s; repeat time, 1. Finally, the data were normalized as described for the genetic reporter assay [40].

### 4.11. MTT Assay and Western Blot Analyses

Cell viability was measured by MTT (3-(4,5-Dimethylthiazol-2-yl)-2,5-Diphenyltetrazolium Bromide) (Invitrogen). Absorbance was quantified with a ThermoFisher Spectrophotometer 1000 (Molecular Devices, Inc.) at a wavelength of 540 nm. Crizotinib (PF-02341066, Selleck Chemicals) (0.25 µM and 2 µM for H3122 and H2228 cells respectively) was used as a positive control.

Cells were lysed in buffer containing 150 mM NaCl, 1% NP-40, 0.5% deoxycholic acid sodium salt, 0.1% SDS, 25 mM Tris-HCL (pH 7.4), and protease inhibitor cocktail set III (Wako Pure Chemical Industries, Japan). Proteins were separated by 9% and 11% SDS-PAGE for blotting with ALK and β-actin antibodies, respectively, and then transferred onto a PVDF membrane (Millipore, Bedford, MA, USA). Proteins were detected by immunoblotting using ECL Select detection reagent (GE Healthcare, Italy). Anti-ALK antibody (Cell Signaling Technology) was used at a dilution of 1:1000. Anti-β–actin antibody (Proteintech) was used at a dilution of 1:2000.

### 4.12. Bioinformatics and Data Analysis

The relationship between *ALK* expression and patient prognosis in lung adenocarcinoma was retrieved from the PrognoScan cancer microarray database [41]. The genetic fusions of ALK with other proteins involved in different types of cancers were obtained from the TCGA PanCancer Atlas through cBioPortal [42,43]. Statistical analyses were performed in MS Excel (version 2016) or GraphPad Prism version 8.0 (GraphPad Software, San Diego, CA, USA). All quantitative data are represented as means ± SEM. A two-tailed Student’s *t*-test was used to evaluate the significance of comparisons between two groups. A *p*-value of 0.05 was considered statistically significant.

## 5. Conclusions

In this study, we determined the half-life of CRISPR Cas13a mRNA and crRNA in human cells. Subsequently, we successfully knocked down firefly luciferase in HEK293T cells (>70%) after optimizing the levels of Cas13a mRNA and guide RNA. In addition, we assessed the effects of the orientation of the crRNA–guide RNA complex, length, and mismatches on Cas13a activity. Knockdown of the oncofusion gene *EML4-ALK* resulted in alteration of cell physiology, reflected by a decrease in viability. Based on our successful robust knockdown of marker genes such as firefly luciferase, mCherry in HEK293T cells, and *EML4-ALK* in H3122 and H2228 lung cancer cells, we speculated that this approach could be used to knock down other transgenes or genes of interest (or RNA of interest, e.g., COVID-19 RNA or noncoding RNA) that affect the biological functions of cells.

## Figures and Tables

**Table 1:** Genetic fusion of ALK with other mRNAs involved in different types of cancers, based on data retrieved from the TCGA PanCancer Atlas using cBioPortal (10,953 patients/10,967 samples).

Fusion	Copy Number	Cancer Type	Patients (*n*)
*EML4-ALK*	diploid	Lung adenocarcinoma	103
*EML4-ALK*	gain	Lung adenocarcinoma	49
*EML4-ALK*	diploid	Papillary renal cell carcinoma	18
*EML4-ALK*	shallowdel	Papillary thyroid cancer	18
*STRN-ALK*	shallowdel	Papillary renal cell carcinoma	44
*STRN-ALK*	deepdel	Papillary thyroid cancer	7
*TPM1-ALK*	diploid	Bladder urothelial carcinoma	89
*PPP4R3B-ALK*	shallowdel	Rectal adenocarcinoma	136
*ACTG2-ALK*	amp	Leiomyosarcoma	51
*ALK-STK39*	shallowdel	Cutaneous melanoma	171
*KCNQ5-ALK*		Cutaneous melanoma	506
*MALAT1-ALK*	diploid	Papillary thyroid cancer	31
*GTF2IRD1-ALK*	diploid	Papillary thyroid cancer	12
*ALK-GALNT14*	gain	Uterine serous carcinoma/uterine papillary serous carcinoma	124
*ALK-SCEL*	gain	Uterine endometrioid carcinoma	156

## Supplementary Materials

The following are available online at https://www.mdpi.com/1422-0067/21/23/8904/s1.

## Abbreviations

CRISPR	Clustered Regularly Interspaced Short Palindromic Repeats
crRNA	CRISPR RNA
*EML4-ALK*	*Echinoderm Microtubule-associated protein-like 4-Anaplastic Lymphoma Kinase*
RNAi	Ribonucleic Acid interference
FLuc	Firefly Luciferase
ActD	Actinomycin D
