# Supplementary material for: Effective RNA Knockdown Using CRISPR-Cas13a and Molecular Targeting of the EML4-ALK Transcript in H3122 Lung Cancer Cells
Source: Int J Mol Sci. 2020 Nov 24;21(23):8904. doi: 10.3390/ijms21238904 (PMC7727695; doi:10.3390/ijms21238904)
Supplement: Supplementary file 1 [file ijms-21-08904-s001.pdf]

Article

Effective RNA knockdown using CRISPR-Cas13a and molecular targeting of the *EML4-ALK* transcript in H3122 lung cancer cells

Saifullah [1], Matomo Sakari [1], Takeshi Suzuki [3,4], Seiji Yano [5,6], and Toshifumi Tsukahara [1,2,] *

[1] Area of Bioscience and Biotechnology, School of Materials Science, Japan Advanced Institute of Science and Technology (JAIST), 1–1 Asahidai, Nomi, Ishikawa 923–1292, Japan; saifullah@jaist.ac.jp (S.); m-sakari@jaist.ac.jp (M.S.);

[2] Division of Transdisciplinary Science, Japan Advanced Institute of Science and Technology, 1–1 Asahidai, Nomi city, Ishikawa 923–1292, Japan;

[3] Division of Functional Genomics, Cancer Research Institute, Kanazawa University, Kakuma-machi, Kanazawa, 920-1192, Ishikawa, Japan; suzuki-t@staff.kanazawa-u.ac.jp (T.S.);

[4] Molecular Therapeutic Target Research Unit, Institute for Frontier Science Initiative, Kanazawa University, Kamuma-cho, Kanazawa 920-1192, Japan;

[5] Division of Medical Oncology, Cancer Research Institute, Kanazawa University, 13-1 Takara-machi, Kanazawa 920-0934, Japan; syano@staff.kanazawa-u.ac.jp (S.Y.);

[6] Nano Life Science Institute, Kanazawa University, Kakuma-machi, Kanazawa 920-1192, Japan;

* Correspondence: tukahara@jaist.ac.jp; Tel.: +81-761-51-1640 (T.T.)

Supplementary Material

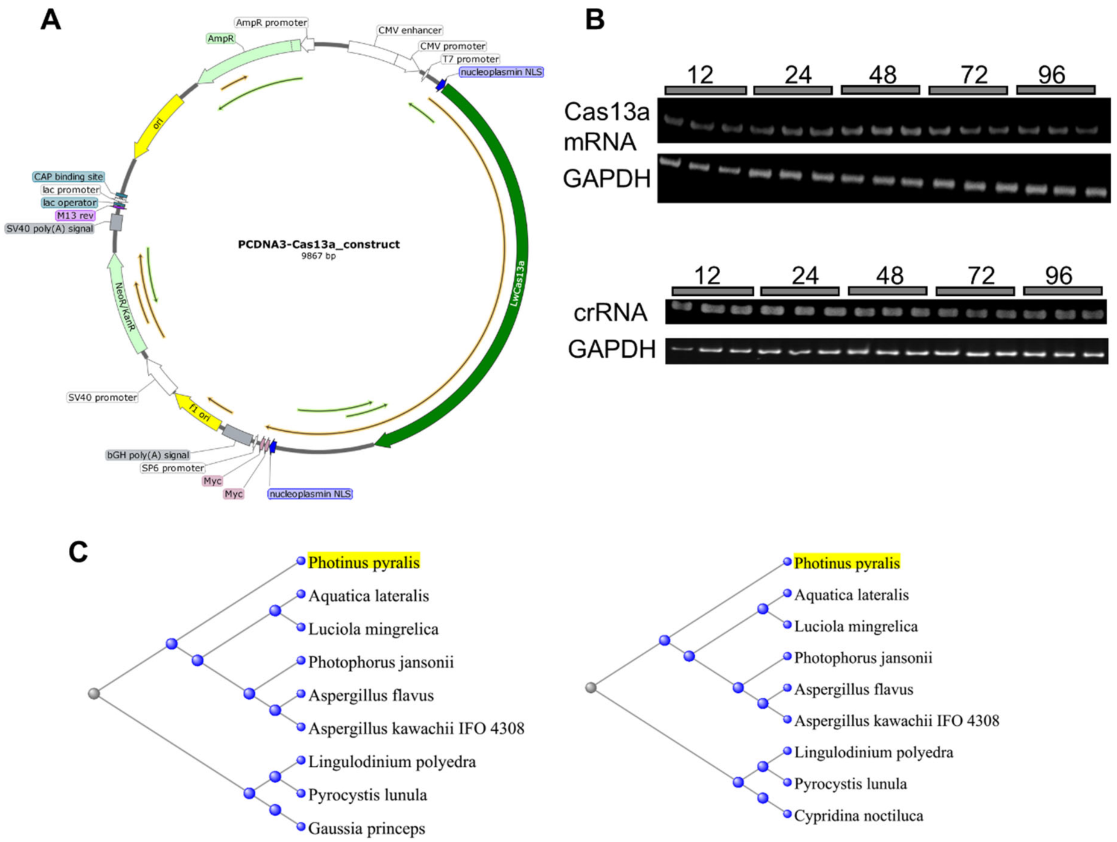

Figure S1. (**A**) Circular map of pcDNA3-Cas13a construct vector plasmid. (**B**) RT-PCR gel images of Cas13a and crRNA guide. (**C**) Phylogenic tree of Luciferin originated from various species. Identity of Firefly luciferase protein with gaussia and cypridinia is 29.03% and 27.45% respectively.

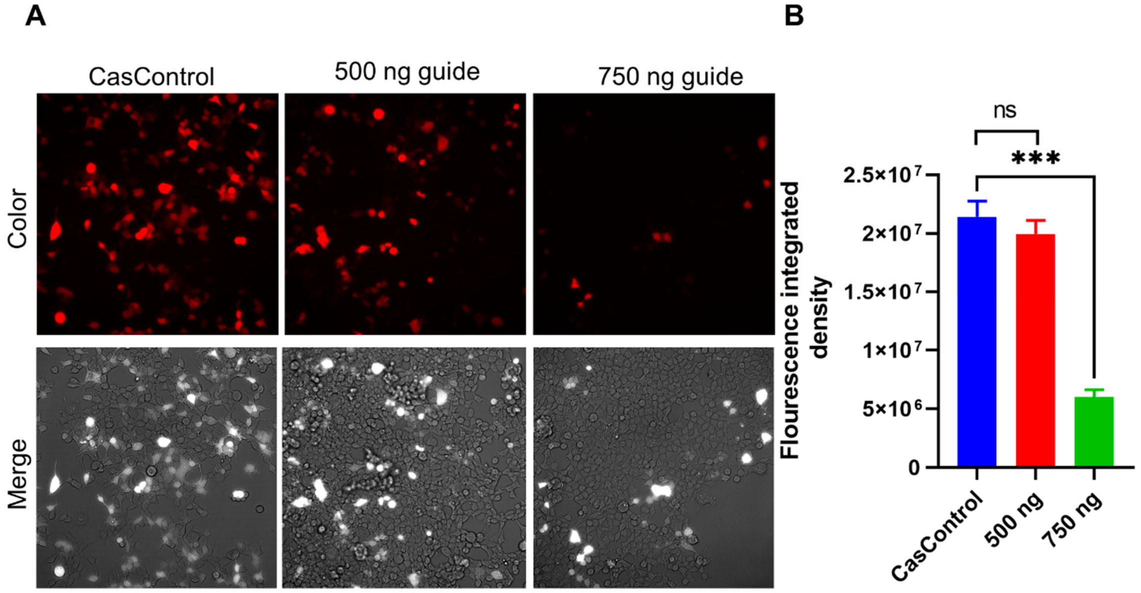

Figure 2. mCherry mRNA knockdown by Cas13a. (**A**) Fluorescence images (20×) of mCherry knockdown at two guide RNA concentrations; (**B**) Graphical representative of A, ***$p < 0.005$.

Supplementary Material

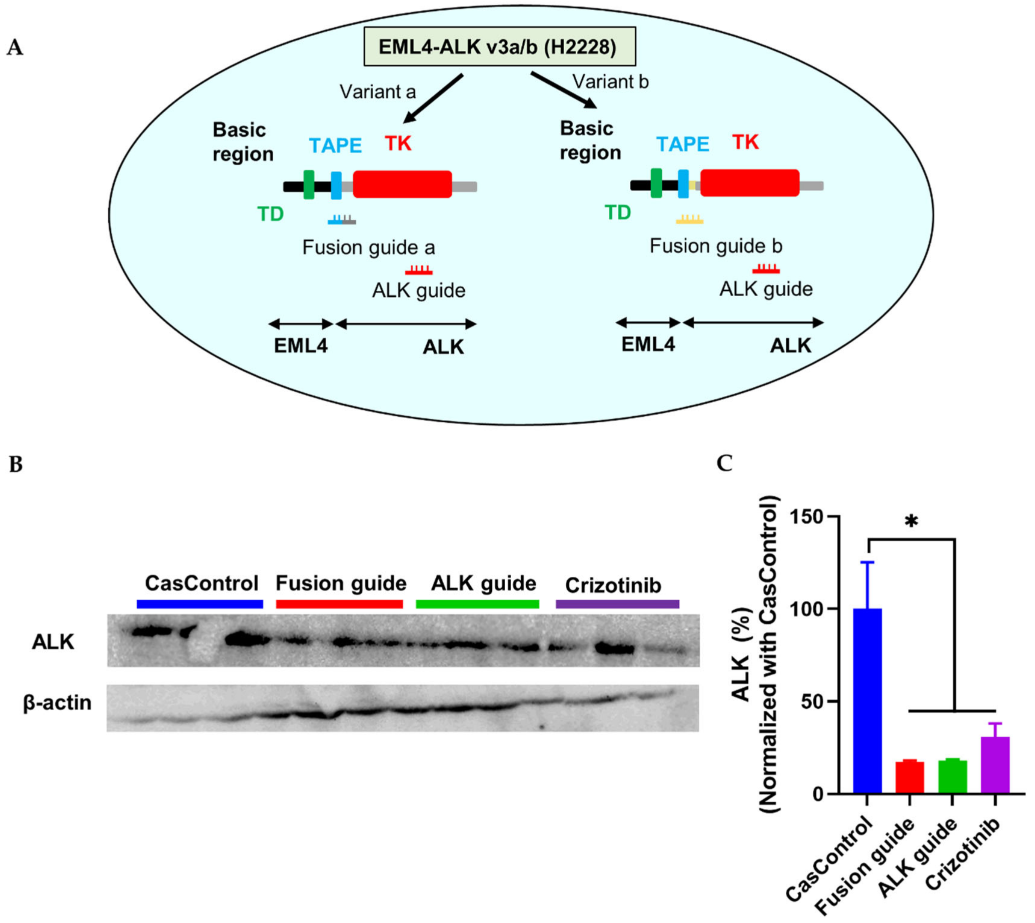

Figure S3. *EML4-ALK* knockdown using Cas13a in H2228 cells. (**A**) Graphical representation of *EML4-ALK* v3a/b oncofusion with the associated functional domain and guide RNA location in H2228 cells. Left side: TD, trimerisation domain; TAPE, tandem atypical propeller domain; HELP, hydrophobic motif in EML proteins; and TK, tyrosine kinase domain. Fusion guide a/b, *EML4-ALK* target guide at the fusion point of *EML4* and *ALK* in variant 3a/3b; ALK guide, *EML4-ALK* target guide in the TK domain. (**B**) Western blot analysis of ALK in H2228 cells treated with Cas13a and indicated guide RNAs or crizotinib (as a control drug) for 72 h. Total β-actin was used as a loading control. ALK protein was down-regulated over 80% in both indicated guide RNAs. (**C**) Graphical representative of **B,** *$p < 0.05$.

Table 1. Oligo sequences used for PCR amplification reaction.

Target	Oligos (5′ to 3′)
	mCherry
mCherry_Fwd	cccggatccCACCATGGTGAGCAAGGGCGA
mCherry_Rev	ggggaattcTTACTTGTACAGCTCGTCCATGC
	pcDNA3-Cas13a
Cas13a_Fwd	gggctcgagTAGAGCGCTGCCACCATGAA
Cas13a_Rev	gggtctagattacatttcattcaagtcctcttcagaaatgagcttttgctccatttcattc aagtcctcttcagaaatgagcttttgctcggtaccTTTCTTCTTCTTAGCC TGTCCAGC
pcDNA3_Fwd	aggacttgaatgaaatgtaatctagAGGGCCCTATTCTATAGTGTCACC
pcDNA3_Rev	cttcatggtggcagcgctctactcgagCTCGAGCGGCCGCCAGTGTGAT

Supplementary Material

Table S2. crRNA and guide RNA sequences

Target RNA name	cDNA of guide RNA (5′ to 3′)	Length (bp)	Reference
Non-targeting guide	TTTACAACGTCGTGACTGGGAAAACCCT	28	[16]
crRNA (or DR)	GATTTAGACTACCCCAAAAACGAAGGGGA CTAAAAC	36	[16]
Luc guide-1	CGAGAATCTCACGCAGGCAGTTCTATGAGG	30	custom
Luc guide-2	GTAATCCTGAAGGCTCCTCAGAAACAGCTC	30	custom
	**Length guide**		
Luc 12 guide	GTAATCCTGAAG	12	custom
Luc 18 guide	GTAATCCTGAAGGCTCCT	18	custom
Luc 24 guide	GTAATCCTGAAGGCTCCTCAGAAA	24	custom
Luc 28 guide	GTAATCCTGAAGGCTCCTCAGAAACAGC	28	custom
Luc 30 guide	GTAATCCTGAAGGCTCCTCAGAAACAGCTC	30	custom
Luc 40 guide	GTAATCCTGAAGGCTCCTCAGAAACAGCTC TTCTTCAAAT	40	custom
Luc 50 guide	GTAATCCTGAAGGCTCCTCAGAAACAGCTC TTCTTCAAA TCTATACATTA	50	custom
	**Mismatch guide**		
Luc WT	GTAATCCTGAAGGCTCCTCAGAAACAGC	28	custom
Luc M1	ATAATCCTGAAGGCTCCTCAGAAACAGC	28	custom
Luc M2	GCAATCCTGAAGGCTCCTCAGAAACAGC	28	custom
Luc M3	GTGATCCTGAAGGCTCCTCAGAAACAGC	28	custom
Luc M4	GTAATCCTGAAGGATCCTCAGAAACAGC	28	custom
Luc M5	GTAATCCTGAAGGCCCCTCAGAAACAGC	28	custom
Luc M6	GTAATCCTGAAGGCTACTCAGAAACAGC	28	custom
Luc M7	GTAATCCTGAAGGCTCCTCAGAAACGGC	28	custom
Luc M8	GTAATCCTGAAGGCTCCTCAGAAACAAC	28	custom
Luc M9	GTAATCCTGAAGGCTCCTCAGAAACAGA	28	custom
	**mCherry**		
mCherry guide	CGAAGTTCATCACGCGCTCCCACTTGAA	28	custom
	**EML4-ALK guide**		
ALK guide	CTGGCAGCAATGTCTCGGTGGATGAAGTGG	30	Custom
Fusion guide (H3122)	GCTTCCGGCGGTACACTTTAGGTCC	25	Custom
Fusion guide a (H2228)	TCCGGCGGTACACTTGGTTGATGATGAC	28	Custom
Fusion guide b (H2228	TTTTCGCGAGTTGACATTTTTGCTTGG	27	Custom

Supplementary Material

Table S3. Primers sequences used for sanger sequencing

Target	Sequences (5′ to 3′)
**PCDNA3-Cas13a**	
T7_forward primer_seqF1	TAATACGACTCACTATAGGG
Cas13a_SeqF2	GAAGATCAACGAGAACAACG
Cas13a_SeqF3	AGGCCTTCCTGAGAAACAT
Cas13a_Seq4	AGTTCCTGAACAAGTTCGT
Cas13a_SeqF5	AAGTTCCTGGACTTCAACG
Cas13a_SeqF6	ACATCCGGAACTACATTGC
BGH-rev	TAGAAGGCACAGTCGAGG
pcDNA_SeqF1	AGAGGGCCCTATTCTATAGTG
pcDNA_SeqF2	GTGTGTCAGTTAGGGTGTG
pcDNA_SeqF3	ATACGCTTGATCCGGCTA
pcDNA_SeqF4	TCATAGCTGTTTCCTGTGTG
pcDNA_SeqF5	GATTAGCAGAGCGAGGTAT
pcDNA_SeqF6	AACGATCAAGGCGAGTTAC
pcDNA_SeqF7	GCTTGACCGACAATTGCAT
**pCS2+crRNA-guide RNA**	
Sp6_fwd	GTGCCTAATGGGAGGTCT

Table S4. Primers sequences used for RT-PCR and qRT-PCR

Target gene	Sequences (5′ to 3′)	Product length (bp)	Assay name
hGAPDH	Fwd: GACAGTCAGCCGCATCTTC<br>Rev: GTTCACACCCATGACGAACAT	464	RNA stability; RT-PCR
	Fwd: GACAGTCAGCCGCATCTTC<br>Rev: ACTCCGACCTTCACCTTCC	79	qRT-PCR
h18S rRNA	Fwd: GGCCCTGTAATTGGAATGAGTC<br>Rev: CCAAGATCCAACTACGAGCTT	146	RNA stability; qRT-PCR
Cas13a_HEPN	Fwd: GGACAACAAGAACGAGATCGAG<br>Rev: GCTGGGGGCGATATTCTTGAA	136	RNA stability; qRT-PCR/RT-PCR
crRNA-guideRNA	Fwd: ATTTAGACTACCCCAAAAACGAAGG<br>Rev: CAGAGCTGTTTCTGAGGAGCC	70	
EML4	Fwd: CTGCTGCAAGTACTTCTGATGTTC<br>Rev: CTTCAGAGATTGCAAGACGCCTC	132	Expression and Knockdown; qRT-PCR
ALK	Fwd: ACATTGCCTGTGGCTGTCAG<br>Rev: ACTTAACTGGCAGCATGGCACA	189	

Supplementary Material

Sanger sequences of pcDNA3-Cas13a construct

cggatcgggagatctcccgatcccctatggtgcactctcagtacaatctgctctgatgccgcatagttaagccagtatctgctccctgcttgtgtgttgg
aggtcgctgagtagtgcgcgagcaaaatttaagctacaacaaggcaaggcttgaccgacaattgcatgaagaatctgcttagggttaggcgttttgc
gctgcttcgcgatgtacgggccagatatacgcgttgacattgattattgactagttattaatagtaatcaattacggggtcattagttcatagcccatata
tggagttccgcgttacataacttacggtaaatggcccgcctggctgaccgcccaacgacccccgcccattgacgtcaataatgacgtatgttcccatag
taacgccaatagggactttccattgacgtcaatgggtggagtatttacggtaaactgcccacttggcagtacatcaagtgtatcatatgccaagtacgc
cccctattgacgtcaatgacggtaaatggcccgcctggcattatgcccagtacatgaccttatgggactttcctacttggcagtacatctacgtattagtc
atcgctattaccatggtgatgcggttttggcagtacatcaatgggcgtggatagcggtttgactcacggggatttccaagtctccaccccattgacgtc
aatgggagtttgttttggcaccaaaatcaacgggactttccaaaatgtcgtaacaactccgccccattgacgcaaatgggcggtaggcgtgtacggt
gggaggtctatataagcagagctctctggctaactagagaacccactgcttactggcttatcgaaattaatacgactcactatagggagacccaagct
tggtaccgagctcggatccactagtaacggccgccagtgtgctggaattctgcagatatccatcacactggcggccgCTCGAGctcgagtaga
gcgctgccaccATGaagcgacctgccgccacaaagaaggctggacaggctaagaagaagaaaggatccatgaaagtgaccaaggtcgacgg
CATCAGCCACAAGAAGTACATCGAAGAGGGCAAGCTCGTGAAGTCCACCAGCGAGGAAAAC
CGGACCAGCGAGAGACTGAGCGAGCTGCTGAGCATCCGGCTGGACATCTACATCAAGAACC
CCGACAACGCCTCCGAGGAAGAGAACCGGATCAGAAGAGAGAACCTGAAGAAGTTCTTTAG
CAACAAGGTGCTGCACCTGAAGGACAGCGTGCTGTATCTGAAGAACCGGAAAGAAAAGAAC
GCCGTGCAGGACAAGAACTATAGCGAAGAGGACATCAGCGAGTACGACCTGAAAAACAAG
AACAGCTTCTCCGTGCTGAAGAAGATCCTGCTGAACGAGGACGTGAACTCTGAGGAACTGGA
AATCTTTCGGAAGGACGTGGAAGCCAAGCTGAACAAGATCAACAGCCTGAAGTACAGCTTC
GAAGAGAACAAGGCCAACTACCAGAAGATCAACGAGAACAACGTGGAAAAAGTGGGCGGC
AAGAGCAAGCGGAACATCATCTACGACTACTACAGAGAGAGCGCCAAGCGCAACGACTACA
TCAACAACGTGCAGGAAGCCTTCGACAAGCTGTATAAGAAAGAGGATATCGAGAAACTGTTT
TTCCTGATCGAGAACAGCAAGAAGCACGAGAAGTACAAGATCCGCGAGTACTATCACAAGA
TCATCGGCCGGAAGAACGACAAAGAGAACTTCGCCAAGATTATCTACGAAGAGATCCAGAA
CGTGAACAACATCAAAGAGCTGATTGAGAAGATCCCCGACATGTCTGAGCTGAAGAAAAGC
CAGGTGTTCTACAAGTACTACCTGGACAAAGAGGAACTGAACGACAAGAATATTAAGTACG
CCTTCTGCCACTTCGTGGAAATCGAGATGTCCCAGCTGCTGAAAAACTACGTGTACAAGCGG
CTGAGCAACATCAGCAACGATAAGATCAAGCGGATCTTCGAGTACCAGAATCTGAAAAAGC
TGATCGAAAACAAACTGCTGAACAAGCTGGACACCTACGTGCGGAACTGCGGCAAGTACAA
CTACTATCTGCAAGTGGGCGAGATCGCCACCTCCGACTTTATCGCCCGGAACCGGCAGAACG
AGGCCTTCCTGAGAAACATCATCGGCGTGTCCAGCGTGGCCTACTTCAGCCTGAGGAACATC
CTGGAAACCGAGAACGAGAACGGTATCACCGGCCGGATGCGGGGCAAGACCGTGAAGAAC
AACAAGGGCGAAGAGAAATACGTGTCCGGCGAGGTGGACAAGATCTACAATGAGAACAAG
CAGAACGAAGTGAAAGAAAATCTGAAGATGTTCTACAGCTACGACTTCAACATGGACAACA
AGAACGAGATCGAGGACTTCTTCGCCAACATCGACGAGGCCATCAGCAGCATCAGACACGG
CATCGTGCACTTCAACCTGGAACTGGAAGGCAAGGACATCTTCGCCTTCAAGAATATCGCCC
CCAGCGAGATCTCCAAGAAGATGTTTCAGAACGAAATCAACGAAAAGAAGCTGAAGCTGAA
AATCTTCAAGCAGCTGAACAGCGCCAACGTGTTCAACTACTACGAGAAGGATGTGATCATCA
AGTACCTGAAGAATACCAAGTTCAACTTCGTGAACAAAAACATCCCCTTCGTGCCCAGCTTC
ACCAAGCTGTACAACAAGATTGAGGACCTGCGGAATACCCTGAAGTTTTTTTGGAGCGTGCC
CAAGGACAAAGAAGAGAAGGACGCCCAGATCTACCTGCTGAAGAATATCTACTACGGCGAG
TTCCTGAACAAGTTCGTGAAAAACTCCAAGGTGTTCTTTAAGATCACCAATGAAGTGATCAA
GATTAACAAGCAGCGGAACCAGAAAACCGGCCACTACAAGTATCAGAAGTTCGAGAACATC
GAGAAAACCGTGCCCGTGGAATACCTGGCCATCATCCAGAGCAGAGAGATGATCAACAACC
AGGACAAAGAGGAAAAGAATACCTACATCGACTTTATTCAGCAGATTTTCCTGAAGGGCTTC
ATCGACTACCTGAACAAGAACAATCTGAAGTATATCGAGAGCAACAACAACAATGACAACA
ACGACATCTTCTCCAAGATCAAGATCAAAAAGGATAACAAAGAGAAGTACGACAAGATCCT
GAAGAACTATGAGAAGCACAATCGGAACAAAGAAATCCCTCACGAGATCAATGAGTTCGTG
CGCGAGATCAAGCTGGGGAAGATTCTGAAGTACACCGAGAATCTGAACATGTTTTACCTGAT
CCTGAAGCTGCTGAACCACAAAGAGCTGACCAACCTGAAGGGCAGCCTGGAAAAGTACCAG
TCCGCCAACAAAGAAGAAACCTTCAGCGACGAGTTGGAACTGATCAACCTGCTGAACCTGG

Supplementary Material

ACAACAACAGAGTGACCGAGGACTTCGAGCTGGAAGCCAACGAGATCGGCAAGTTCCTGGA
CTTCAACGAAAACAAAATCAAGGACCGGAAAGAGCTGAAAAAGTTCGACACCAACAAGAT
CTATTTCGACGGCGAGAACATCATCAAGCACCGGGCCTTCTACAATATCAAGAAATACGGCA
TGCTGAATCTGCTGGAAAAGATCGCCGATAAGGCCAAGTATAAGATCAGCCTGAAAGAACT
GAAAGAGTACAGCAACAAGAAGAATGAGATTGAAAAGAACTACACCATGCAGCAGAACCT
GCACCGGAAGTACGCCAGACCCAAGAAGGACGAAAAGTTCAACGACGAGGACTACAAAGA
GTATGAGAAGGCCATCGGCAACATCCAGAAGTACACCCACCTGAAGAACAAGGTGGAATTC
AATGAGCTGAACCTGCTGCAGGGCCTGCTGCTGAAGATCCTGCACCGGCTCGTGGGCTACAC
CAGCATCTGGGAGCGGGACCTGAGATTCCGGCTGAAGGGCGAGTTTCCCGAGAACCACTACA
TCGAGGAAATTTTCAATTTCGACAACTCCAAGAATGTGAAGTACAAAAGCGGCCAGATCGTG
GAAAAGTATATCAACTTCTACAAAGAACTGTACAAGGACAATGTGGAAAAGCGGAGCATCT
ACTCCGACAAGAAAGTGAAGAAACTGAAGCAGGAAAAAAAGGACCTGTACATCCGGAACT
ACATTGCCCACTTCAACTACATCCCCCACGCCGAGATTAGCCTGCTGGAAGTGCTGGAAAAC
CTGCGGAAGCTGCTGTCCTACGACCGGAAGCTGAAGAACGCCATCATGAAGTCCATCGTGGA
CATTCTGAAAGAATACGGCTTCGTGGCCACCTTCAAGATCGGCGCTGACAAGAAGATCGAAA
TCCAGACCCTGGAATCAGAGAAGATCGTGCACCTGAAGAATCTGAAGAAAAAGAAACTGAT
GACCGACCGGAACAGCGAGGAACTGTGCGAACTCGTGAAAGTCATGTTCGAGTACAAGGCC
CTGGAAGGAGGAGGTGGAAGCGGAGGAGGAGGAAGCGGAGGAGGAGGTAGCGTGAGTAAA
GGTGAAGAACTCTTCACTGGAGTAGTGCCCATTCTGGTAGAGCTTGATGGAGATGTAAATGG
ACATAAATTCTCCGTCAGGGGCGAAGGCGAAGGGGACGCCACGAATGGTAAGCTGACTCTG
AAATTCATCTGTACGACGGGCAAACTGCCCGTCCCATGGCCTACACTCGTAACGACCCTCAC
CTACGGCGTGCAATGCTTTTCTCGATATCCCGACCACATGAAACAGCATGACTTTTTCAAGTC
TGCAATGCCTGAAGGTTATGTTCAAGAAAGGACCATCAGCTTTAAGGATGATGGTACATATA
AAACCCGAGCCGAGGTTAAATTTGAAGGGGACACTCTGGTTAATCGAATTGAACTGAAAGGT
ATTGATTTTAAGGAGGACGGTAACATACTGGGGCACAAGTTGGAGTACAACTTTAACAGCCA
TAATGTGTATATTACCGCTGATAAGCAGAAAAATGGGATAAAGGCCAACTTTAAGATCCGAC
ATAATGTCGAAGATGGTAGTGTTCAACTGGCTGATCATTACCAACAAAATACGCCCATCGGA
GATGGACCTGTACTCTTGCCTGACAATCATTATCTCTCCACGCAATCAAAGCTTTCCAAGGAC
CCAAACGAAAAGAGAGATCACATGGTCCTTCTGGAATTTGTGACTGCCGCAGGCATCactctcggt
atggatgagctgtacaagggatccaagcgacctgccgccacaaagaaggctggacaggctaagaagaagaaaggtaccGAGCAAAAG
CTCATTTCTGAAGAGGACTTGAATGAAATGGAGCAAAAGCTCATTTCTGAAGAGGACTTGAA
TGAAATG*taa*tctagagggccctattctatagtgtcacctaaatgctagagctcgctgatcagcctcgactgtgccttctagttgccagccatctgt
tgtttgcccctcccccgtgccttccttgaccctggaaggtgccactcccactgtcctttcctaataaaatgaggaaattgcatcgcattgtctgagtaggt
gtcattctattctggggggtggggtggggcaggacagcaagggggaggattgggaagacaatagcaggcatgctggggatgcggtgggctcta
tggcttctgaggcggaaagaaccagctggggctctagggggtatccccacgcgccctgtagcggcgcattaagcgcggcgggtgtggtggttac
gcgcagcgtgaccgctacacttgccagcgccctagcgcccgctcctttcgctttcttcccttcctttctcgccacgttcgccggctttccccgtcaagctct
aaatcgggggctccctttagggttccgatttagtgctttacggcacctcgaccccaaaaaacttgattagggtgatggttcacgtagtgggccatcgcc
ctgatagacggtttttcgccctttgacgttggagtccacgttctttaatagtggactcttgttccaaactggaacaacactcaaccctatctcggtctattct
tttgatttataagggattttgccgatttcggcctattggttaaaaaatgagctgatttaacaaaaatttaacgcgaattaattctgtggaatgtgtgtcagt
tagggtgtggaaagtccccaggctccccagcaggcagaagtatgcaaagcatgcatctcaattagtcagcaaccaggtgtggaaagtccccaggc
tccccagcaggcagaagtatgcaaagcatgcatctcaattagtcagcaaccatagtcccgcccctaactccgcccatcccgcccctaactccgcccag
ttccgcccattctccgccccatggctgactaattttttttatttatgcagaggccgaggccgcctctgcctctgagctattccagaagtagtgaggaggct
tttttggaggcctaggcttttgcaaaaagctcccgggagcttgtatatccattttcggatctgatcaagagacaggatgaggatcgtttcgcatgattga
acaagatggattgcacgcaggttctccggccgcttgggtggagaggctattcggctatgactgggcacaacagacaatcggctgctctgatgccgc
cgtgttccggctgtcagcgcaggggcgcccggttctttttgtcaagaccgacctgtccggtgccctgaatgaactgcaggacgaggcagcgcggct
atcgtggctggccacgacgggcgttccttgcgcagctgtgctcgacgttgtcactgaagcgggaagggactggctgctattgggcgaagtgccgg
ggcaggatctcctgtcatctcaccttgctcctgccgagaaagtatccatcatggctgatgcaatgcggcggctgcatacgcttgatccggctacctgcc
cattcgaccaccaagcgaaacatcgcatcgagcgagcacgtactcggatggaagccggtcttgtcgatcaggatgatctggacgaagagcatcag
gggctcgcgccagccgaactgttcgccaggctcaaggcgcgcatgcccgacggcgaggatctcgtcgtgacccatggcgatgcctgcttgccgaa
tatcatggtggaaaatggccgcttttctggattcatcgactgtggccggctgggtgtggcggaccgctatcaggacatagcgttggctacccgtgata
ttgctgaagagcttggcggcgaatgggctgaccgcttcctcgtgctttacggtatcgccgctcccgattcgcagcgcatcgccttctatcgccttcttga

Supplementary Material

cgagttcttctgagcgggactctggggttcgaaatgaccgaccaagcgacgcccaacctgccatcacgagatttcgattccaccgccgccttctatga
aaggttgggcttcggaatcgttttccgggacgccggctggatgatcctccagcgcggggatctcatgctggagttcttcgcccaccccaacttgtttat
tgcagcttataatggttacaaataaagcaatagcatcacaaatttcacaaataaagcatttttttcactgcattctagttgtggtttgtccaaactcatcaat
gtatcttatcatgtctgtataccgtcgacctctagctagagcttggcgtaatcatggtcatagctgtttcctgtgtgaaattgttatccgctcacaattccac
acaacatacgagccggaagcataaagtgtaaagcctggggtgcctaatgagtgagctaactcacattaattgcgttgcgctcactgcccgctttccag
tcgggaaacctgtcgtgccagctgcattaatgaatcggccaacgcgcggggagaggcggtttgcgtattgggcgctcttccgcttcctcgctcactg
actcgctgcgctcggtcgttcggctgcggcgagcggtatcagctcactcaaaggcggtaatacggttatccacagaatcaggggataacgcagga
aagaacatgtgagcaaaaggccagcaaaaggccaggaaccgtaaaaaggccgcgttgctggcgtttttccataggctccgcccccctgacgagca
tcacaaaaatcgacgctcaagtcagaggtggcgaaacccgacaggactataaagataccaggcgtttccccctggaagctccctcgtgcgctctcct
gttccgaccctgccgcttaccggatacctgtccgcctttctcccttcgggaagcgtggcgctttctcatagctcacgctgtaggtatctcagttcggtgta
ggtcgttcgctccaagctgggctgtgtgcacgaaccccccgttcagcccgaccgctgcgccttatccggtaactatcgtcttgagtccaacccggtaa
gacacgacttatcgccactggcagcagccactggtaacaggattagcagagcgaggtatgtaggcggtgctacagagttcttgaagtggtggccta
actacggctacactagaagaacagtatttggtatctgcgctctgctgaagccagttaccttcggaaaaagagttggtagctcttgatccggcaaacaa
accaccgctggtagcggtggtttttttgtttgcaagcagcagattacgcgcagaaaaaaaggatctcaagaagatcctttgatcttttctacggggtct
gacgctcagtggaacgaaaactcacgttaagggattttggtcatgagattatcaaaaaggatcttcacctagatccttttaaattaaaaatgaagtttta
aatcaatctaaagtatatatgagtaaacttggtctgacagttaccaatgcttaatcagtgaggcacctatctcagcgatctgtctatttcgttcatccatag
ttgcctgactccccgtcgtgtagataactacgatacgggagggcttaccatctggccccagtgctgcaatgataccgcgagacccacgctcaccggct
ccagatttatcagcaataaaccagccagccggaagggccgagcgcagaagtggtcctgcaactttatccgcctccatccagtctattaattgttgccg
ggaagctagagtaagtagttcgccagttaatagtttgcgcaacgttgttgccattgctacaggcatcgtggtgtcacgctcgtcgtttggtatggcttca
ttcagctccggttcccaacgatcaaggcgagttacatgatcccccatgttgtgcaaaaaagcggttagctccttcggtcctccgatcgttgtcagaagt
aagttggccgcagtgttatcactcatggttatggcagcactgcataattctcttactgtcatgccatccgtaagatgcttttctgtgactggtgagtactca
accaagtcattctgagaatagtgtatgcggcgaccgagttgctcttgcccggcgtcaatacgggataataccgcgccacatagcagaactttaaaag
tgctcatcattggaaaacgttcttcggggcgaaaactctcaaggatcttaccgctgttgagatccagttcgatgtaacccactcgtgcacccaactgatc
ttcagcatcttttactttcaccagcgtttctgggtgagcaaaaacaggaaggcaaaatgccgcaaaaaagggaataagggcgacacggaaatgttg
aatactcatactcttcctttttcaatattattgaagcatttatcagggttattgtctcatgagcggatacatatttgaatgtatttagaaaaataaacaaata
ggggttccgcgcacatttccccgaaaagtgccacctgacgtcga.
